# Theophylline

**DOI:** 10.3390/ph3030725

**Published:** 2010-03-18

**Authors:** Peter J. Barnes

**Affiliations:** National Heart and Lung Institute, Imperial College, London, UK; Email: p.j.barnes@imperial.ac.uk; Tel.: +44-207-351-8174; Fax: +44-207-351-5675.

**Keywords:** methylxanthine, phosphodiesterase, adenosine receptor, histone deacetylase, bronchodilatation, inflammation, immunomodulation, plasma concentration, drug interaction

## Abstract

Theophylline (3-methyxanthine) has been used to treat airway diseases for over 70 years. It was originally used as a bronchodilator but the relatively high doses required are associated with frequent side effects, so its use declined as inhaled β_2_-agonists became more widely used. More recently it has been shown to have anti-inflammatory effects in asthma and COPD at lower concentrations. The molecular mechanism of bronchodilatation is inhibition of phosphodiesterase(PDE)3 and PDE4, but the anti-inflammatory effect may be due to histone deacetylase (HDAC) activation, resulting in switching off of activated inflammatory genes. Through this mechanism theophylline also reverses corticosteroid resistance and this may be of particular value in severe asthma and COPD where HDAC2 activity is markedly reduced. Theophylline is given systemically (orally as slow-release preparations for chronic treatment and intravenously for acute exacerbations of asthma) and blood concentrations are determined mainly by hepatic metabolism, which may be increased or decreased in several diseases and by concomitant drug therapy. Theophylline is now usually used as an add-on therapy in asthma patients not well controlled on inhaled corticosteroids and in COPD patients with severe disease not controlled by bronchodilator therapy. Side effects are related to plasma concentrations and include nausea, vomiting and headaches due to PDE inhibition and at higher concentrations to cardiac arrhythmias and seizures due to adenosine A_1_-receptor antagonism.

## 1. Introduction

Theophylline remains one of the most widely prescribed drugs for the treatment of asthma and COPD world-wide, since it is inexpensive and widely available. In many industrialized countries, however, theophylline has become a third-line treatment that is only used in poorly controlled patients as an add-on therapy. This has been reinforced by various national and international guidelines on asthma and COPD therapy. Some have even questioned whether theophylline is indicated in any patients with asthma [1], although others have emphasized the special beneficial effects of theophylline which still give it an important place in management of asthma [2]. The frequency of side-effects at the previously recommended doses and the relatively low efficacy of theophylline have recently led to reduced usage, since inhaled β_2_-agonists are far more effective as bronchodilators and inhaled corticosteroids have a greater anti-inflammatory effect. Despite the fact that theophylline has been used in asthma therapy for over 70 years, there is still considerable uncertainty about its molecular mode of action in asthma and its logical place in therapy. Recently novel mechanisms of action that may account for the effectiveness of theophylline in severe asthma have been elucidated [3]. Because of problems with side effects, there have been attempts to improve on theophylline and recently there has been increasing interest in selective phosphodiesterase (PDE) inhibitors, which have the possibility of improving the beneficial and reducing the adverse effects of theophylline.

## 2. Chemistry

Theophylline is a methylxanthine, similar in structure to the common dietary xanthines caffeine and theobromine. Several substituted derivatives have been synthesized, but none has any advantage over theophylline [2]. The 3-propyl derivative, enprofylline, is more potent as a bronchodilator and may have fewer toxic effects; however, its clinical development was halted because of hepatic toxicity problems [4]. Many salts of theophylline have also been marketed, the most common being aminophylline, the ethylene diamine salt used to increase solubility at neutral pH, so that intravenous administration is possible. Other salts, such as choline theophyllinate, do not have any advantage and others, such as acepifylline, are virtually inactive. Doxofylline (7-(1,3-dioxalan-2-yl-methyl) theophylline) has similar efficacy to theophylline but has less effect on adenosine receptors so fewer cardiovascular and central side effects [5]. It is well tolerated when given twice daily by mouth.

## 3. Molecular Mechanisms of Action

Although theophylline has been in clinical use for more than 70 years, its mechanism of action at a molecular level and its site of action remain uncertain, although there have been important recent advances. Several molecular mechanisms of action have been proposed, many of which appear to occur only at higher concentrations of theophylline than are effective clinically (Table 1).

**Table 1:** Proposed mechanisms of action of theophylline.

Phosphodiesterase inhibition (non-selective) Adenosine receptor antagonism (A_1_-, A_2A_-, A_2B_-receptors) Inhibition of nuclear factor-κB (↓ nuclear translocation) Inhibition of phosphoinositide 3 kinase-δ ↑ Interleukin-10 secretion ↑ Apoptosis of inflammatory cells ↓ poly(ADP-ribose)polymerase-1 (inhibits cell death) ↑ Histone deacetylase activity (↑ efficacy of corticosteroids)

### 3.1. Phosphodiesterase Inhibition

**Figure 1:**
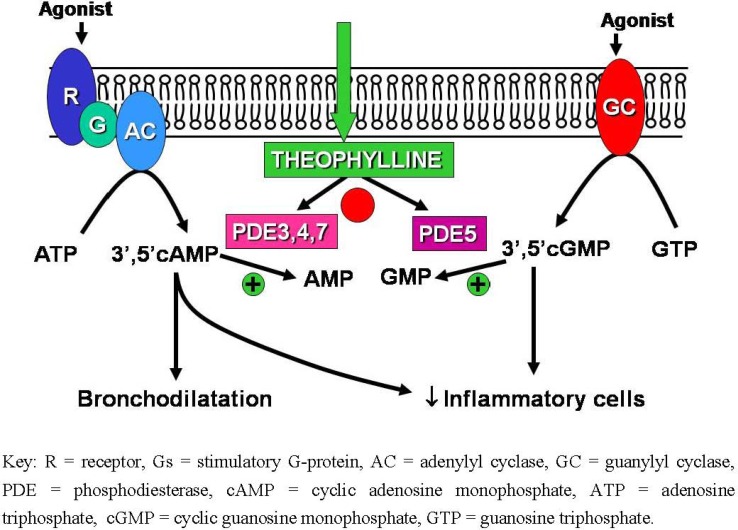
Effect of phosphodiesterase (PDE) inhibitors in the breakdown of cyclic nucleotides in airway smooth muscle and inflammatory cells.

Theophylline is a weak non-selective inhibitor of PDEs, which break down cyclic nucleotides in the cell, thereby leading to an increase in intracellular cyclic 3'5' adenosine monophosphate (AMP) and cyclic 3',5' guanosine monophosphate (GMP) concentrations (Figure 1). However, the degree of inhibition is small at concentrations of theophylline that are therapeutically relevant. Thus total PDE activity in human lung extracts is inhibited by only 5–10% by therapeutic concentrations of theophylline [6]. There is convincing *in vitro* evidence that theophylline relaxes airway smooth muscle by inhibition of PDE activity, but relatively high concentrations are needed for maximal relaxation [7]. Similarly, the inhibitory effect of theophylline on mediator release from alveolar macrophages appears to be mediated by inhibition of PDE activity in these cells [8]. There is no evidence that airway smooth muscle or inflammatory cells concentrate theophylline to achieve higher intracellular than circulating concentrations. Inhibition of PDE should lead to synergistic interaction with β-agonists, but this has not been convincingly demonstrated *in vivo* or in clinical studies; however, this might be because relaxation of airway smooth muscle by β-agonists may involve direct coupling of β-receptors *via* a stimulatory G-protein to the opening of potassium channels, without the involvement of cyclic AMP [9].

At least 11 isoenzyme families of PDE have now been recognized and some (PDE3, PDE4, PDE5) are important in smooth muscle relaxation [10]; however, there is no convincing evidence that theophylline has any greater inhibitory effect on the PDE isoenzymes involved in smooth muscle relaxation. It is possible that PDE isoenzymes may have an increased expression in asthmatic airways, either as a result of the chronic inflammatory process, or as a result of therapy. Elevation of cyclic AMP by ß-agonists may result in increased PDE activity, thus limiting the effect of ß-agonists. Indeed, alveolar macrophages from asthmatic patients appear to have increased PDE activity [11]. This would mean that theophylline might have a greater inhibitory effect on PDE in asthmatic airways than in normal airways. Support for this is provided by the lack of bronchodilator effect of theophylline in normal subjects, compared to a bronchodilator effect in asthmatic patients [12].

Inhibition of PDEs is likely to account for some of the most frequent side effects of theophylline, including nausea and vomiting (PDE4), palpitations and cardiac arrhythmias (PDE3) and headaches (PDE4).

### 3.2. Adenosine Receptor Antagonism

Theophylline is a potent inhibitor of adenosine receptors at therapeutic concentrations. Both A_1_- and A_2_-receptors are inhibited, but theophylline is less effective against A_3_-receptors, suggesting that this could be the basis for its bronchodilator effects [13]. Although adenosine has little effect on normal human airway smooth muscle *in vitro*, it constricts airways of asthmatic patients *via* the release of histamine and leukotrienes, suggesting that adenosine releases mediators from mast cells [14]. The receptor involved appears to be an A_3_- receptor in rat mast cells [15,16], but in humans A_2B_-receptors are involved [17]. Adenosine causes bronchoconstriction in asthmatic subjects when given by inhalation [18]. The mechanism of bronchoconstriction is indirect and involves release of histamine from airway mast cells [14,19]. The bronchoconstrictor effect of adenosine is prevented by therapeutic concentrations of theophylline [18]; however, this only confirms that theophylline is capable of antagonizing the effects of adenosine at therapeutic concentrations, and does not necessarily indicate that this is important for its anti-asthma effect. Adenosine antagonism is likely to account for some of the side effects of theophylline, such as central nervous system stimulation, cardiac arrhythmias (both *via* blockade of A_1_-receptors), gastric hypersecretion, gastroesophageal reflux and diuresis. A novel AMP receptor, P2Y_15_, has been identified which is more potently inhibited by theophylline [20], although the function of these receptors has been questioned.

### 3.3. Interleukin-10 Release

Interleukin(IL)-10 has a broad spectrum of anti-inflammatory effects and there is evidence that its secretion is reduced in asthma and COPD [21]. IL-10 release is increased by theophylline and this effect may be mediated *via* PDE inhibition [22], although this has not been seen at the low doses that are effective in asthma [23].

### 3.4. Effects on Gene Transcription

Theophylline prevents the translocation of the proinflammatory transcription factor nuclear factor-κB (NF-κB) into the nucleus, thus potentially reducing the expression of inflammatory genes in asthma and COPD [24]. Inhibition of NF-κB appears to be due to a protective effect against the degradation of the inhibitory protein I-κBα, so that nuclear translocation of activated NF-κB is prevented [25]. However, these effects are seen at high concentrations and may be mediated by inhibition of PDE. 

### 3.5. Effect on Kinases

Theophylline directly inhibits phosphoinositie-3-kinases, with greatest potency for the PI3K (p110)-δ subtype (IC_50_ 75 μM) [26], a subtype of the enzyme that has been implicated in responses to oxidative stress [27]. However, it is a relatively weak effect against the PI3K-γ subtype (IC_50_ 800 μM), which is involved in chemotactic responses of neutrophils and monocytes. The inhibitory effect of theophylline on PI3K-δ may account for the ability of theophylline to reverse corticosteroid resistance, which may be of critical importance for its clinical effects in severe asthma and COPD [28].

### 3.6. Effects on Apoptosis

Prolonged survival of granulocytes due to a reduction in apoptosis may be important in perpetuating chronic inflammation in COPD. Theophylline promotes inhibits apoptosis in neutrophils *in vitro* [29]. This is associated with a reduction in the anti-apoptotic protein Bcl-2 [30]. This effect is not mediated *via* PDE inhibition, but in neutrophils may be mediated by antagonism of adenosine A_2A_-receptors [31]. Theophylline also induces apoptosis of T-lymphocytes, thus reducing their survival and this effect appears to be mediated *via* PDE inhibition [32]. Theophylline also inhibits the enzyme poly(ADP-ribose)polymerase-1 (PARP-1), which is activated by oxidative stress and leads to a reduction in nicotine adenine diamine levels resulting in an energy crisis that leads to cell death [33]. 

### 3.7. Histone Deacetylase Activation

**Figure 2:**
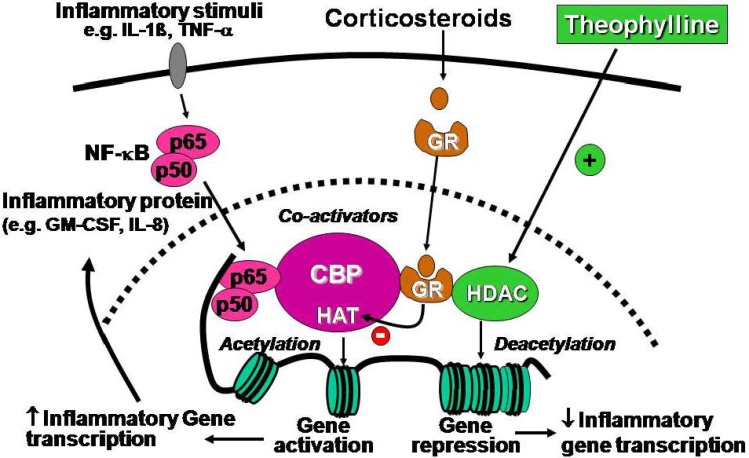
Theophylline directly activates histone deacetylases (HDACs) which deacetylate core histones that have been acetylated by the histone acetyltransferase (HAT) activity of co-activators, such as CREB-binding protein (CBP). This results in suppression of inflammatory genes and proteins, such as granulocyte-macrophage colony stimulating factor (GM-CSF) and interleukin-8 (IL-8) that have been switched on by proinflammatory transcription factors, such as nuclear factor-κB (NF-κB). Corticosteroids also activate HDACs, but through a different mechanism resulting in the recruitment of HDACs to the activated transcriptional complex *via* activation of the glucocorticoid receptors (GR) which function as a molecular bridge. This predicts that theophylline and corticosteroids may have a synergistic effect in repressing inflammatory gene expression.

A novel mechanism of action involving activation of histone deacetylases (HDAC) has been described which, in contrast to the proposed molecular mechanisms discussed above, is seen at therapeutically relevant concentrations [3]. Expression of inflammatory genes is regulated by the balance between histone acetylation and deacetylation [34]. In asthma multiple inflammatory genes are activated through proinflammatory transcription factors, such as NF-κB, leading to histone acetylation and increased transcription. This process is reversed by the recruitment of histone deacetylases (HDAC) to the activated inflammatory gene promoter site within the nucleus. Corticosteroids suppress inflammation by recruiting HDAC2 to activated inflammatory genes, thus switching off their expression [35]. This molecular mechanism is defective in COPD patients as HDAC2 activity and expression is markedly reduced, thus accounting for the steroid resistance of COPD [36]. There is also a defect in HDAC2 function in patients with severe asthma and in asthmatic patients who smoke [37,38]. Theophylline is an activator of HDACs and enhances the anti-inflammatory effect of corticosteroids, as well as reversing steroid resistance in cells from COPD patients [39,40] (Figure 2). 

This action of theophylline is seen at low plasma concentrations (optimally 5 mg/L) and is completely independent of PDE inhibition and adenosine antagonism. The effect of theophylline is reversed by an HDAC inhibitor called trichostatin A and by knocking out HDAC2 using interference RNA [41]. The reason why theophylline selectivey activates HDAC activity is through the inhibition of PI3K-δ, which is activated by oxidative stress and involved in the regulation of HDAC2 activity [28]. This effect of theophylline is seen particularly in the presence of oxidative and nitrative stress and this accounts for why theophylline is effective particularly in severe asthma, where oxidative and nitrative stress are greatest. Increased reactive oxygen species and nitric oxide from increased expression of inducible nitric oxide synthase results in the formation of peroxynitrite radicals. Peroxynitrite is unstable and nitrates tyrosine residues in proteins, which may result in altered protein function. Peroxynitrite is increased in COPD lungs [42,43] and is associated with tyrosine nitration and inactivation of HDAC2 [44]. Theophylline also appears to reduce the formation of peroxynitrite and this provides a further mechanism for increasing HDAC2 function in asthma [45].

## 4. Cellular Effects

**Figure 3:**
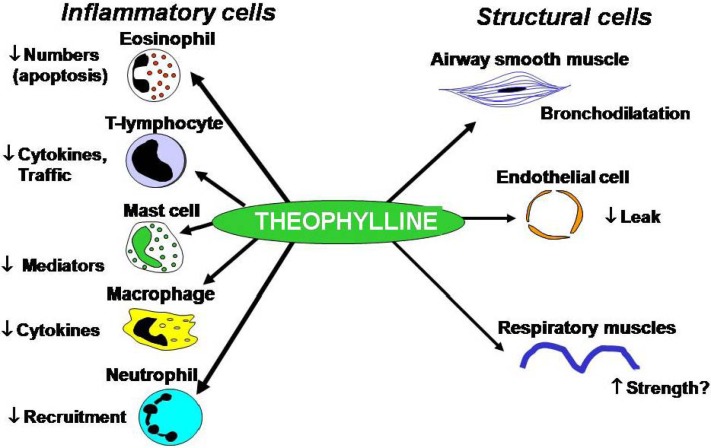
Multiple cellular effects of theophylline.

Theophylline has several cellular effects that may contribute to its clinical efficacy in the treatment of asthma (Figure 3).

### 4.1. Airway Smooth Muscle Effects

The primary effect of theophylline was assumed to be relaxation of airway smooth muscle and *in vitro* studies have shown that it is equally effective in large and small airways [46]. In airways obtained at lung surgery approximately 25% of preparations fail to relax with a ß-agonist, but all relax with theophylline [47]. The molecular mechanism of bronchodilatation is almost certainly related to PDE inhibition, resulting in an increase in cyclic AMP [7]. PDE3 inhibition accounts for most of the bronchodilator effect, with no contribution from PDE4 inhibition in human airway smooth muscle. The bronchodilator effect of theophylline is reduced in human airways by the toxin charybdotoxin, which inhibits large conductance Ca^2+^-activated K^+^ channels (maxi-K channels), suggesting that theophylline opens maxi K channels *via* an increase in cyclic AMP [48]. Theophylline acts as a functional antagonist and inhibits the contractile response of multiple spasmogens. In airways obtained at post-mortem from patients who have died from asthma the relaxant response to β-agonists is reduced, whereas the bronchodilator response to theophylline is no different from that seen in normal airways [49]. Theophylline is a weak bronchodilator at therapeutically relevant concentrations, suggesting that some other target cell may be more relevant for its anti-asthma effect. In human airways the EC_50_ for airway smooth muscle relaxation by theophylline is approximately 1.5 × 10^-4 ^M, which is equivalent to 67 mg/L assuming 60% protein binding [47], that is well above the therapeutic range. However, PDE activity may be increased in asthmatic airways as a result of inflammation so that theophylline may have a greater than expected effect.

*In vivo* intravenous aminophylline has an acute bronchodilator effect in asthmatic patients, which is most likely to be due to a relaxant effect on airway smooth muscle [50]. The bronchodilator effect of theophylline in chronic asthma is small in comparison with β_2_-agonists, however. Several studies have demonstrated a small protective effect of theophylline on histamine, methacholine or exercise challenge [51,52,53,54]. This protective effect does not correlate well with any bronchodilator effect and the protective effect of theophylline may be observed at plasma concentrations of <10 mg/L. These clinical studies suggest that theophylline may have anti-asthma effects that are unrelated to any bronchodilator action.

### 4.2. Anti-inflammatory Effects

There is increasing evidence that theophylline has anti-inflammatory effects in asthma and that these are seen at lower plasma concentrations than are needed for bronchodilatation. Theophylline inhibits mediator release from chopped human lung [55], although high concentrations are necessary and it is likely that this effect involves an increase in cyclic AMP concentration due to PDE inhibition. Theophylline also has an inhibitory effect on superoxide anion release from human neutrophils [56] and inhibits the feedback stimulatory effect of adenosine on neutrophils *in vivo* [57]. At therapeutic concentrations *in vitro* theophylline may *increase* superoxide release *via* an inhibitory effect on adenosine receptors, since endogenous adenosine may normally exert an inhibitory action on these cells [58]. Similar results are also seen in guinea-pig and human eosinophils [59]. At therapeutic concentrations there is an increased release of superoxide anions from eosinophils, which appears to be mediated *via* inhibition of adenosine A_2_-receptors and is mimicked by the adenosine antagonist 8-phenyltheophylline. Inhibition of eosinophil superoxide generation occurs only at high concentrations of theophylline (> 10^-4 ^M) which are likely to inhibit PDE. Similar results have also been obtained in human alveolar macrophages [8]. Macrophages in bronchoalveolar lavage fluid from patients taking theophylline have been found to have a reduced oxidative burst response [60], but there is no reduction in the release of the proinflammatory cytokines TNF–α or GM-CSF [61]. Theophylline inhibits neutrophil chemotaxis *via* inhibition of adenosine A_2A_-receptors and this may be relevant in severe asthma [62].

*In vivo* theophylline inhibits mediator-induced airway microvascular leakage in rodents when given in high doses [63], although this is not seen at therapeutically relevant concentrations [64]. Theophylline has an inhibitory effect on plasma exudation in nasal secretions induced by allergen in patients with allergic rhinitis, although this could be secondary to inhibition of mediator release [65]. Microarray studies in macrophages show that theophylline has the greatest inhibitory effect on IL-13 expression [66].

In allergen challenge studies chronic oral treatment with theophylline reduces the late response to allergen [67]. This has been interpreted as an effect on the chronic inflammatory response and is supported by a reduced infiltration of eosinophils into the airways after allergen challenge following low doses of theophylline [68]. In patients with nocturnal asthma low dose theophylline inhibits the influx of neutrophils and, to a lesser extent, eosinophils seen in the early morning [69]. Chronic treatment with low dose theophylline reduces the numbers of eosinophils in bronchial biopsies, bronchoalveolar lavage and induced sputum of patients with mild asthma [61]. However these effects are less than those expected with low doses of inhaled corticosteroids and there is no reduction of exhaled nitric oxide, indicating a lesser effect on suppression of inflammation than corticosteroids.

In patients with COPD, theophylline reduces the proportion of neutrophils in induced sputum and reduces the concentration of IL-8, suggesting that it may have an anti-inflammatory effect unlike corticosteroids [70,71,72]. Since patients with severe asthma may have increased neutrophils in the airways [73,74], this may provide a mechanism whereby theophylline is effective as an add-on therapy to high doses of inhaled corticosteroids in these patients.

### 4.3. Immunomodulatory Effects

T-lymphocytes are now believed to play a central role in coordinating the chronic inflammatory response in asthma. Theophylline has several actions on T-lymphocyte function, suggesting that it might have an immunomodulatory effect in asthma. Theophylline has a stimulatory effect on suppressor (CD8^+^) T-lymphocytes, which may be relevant to the control of chronic airway inflammation [75,76], and has an inhibitory effect on graft rejection [77]. *In vitro* theophylline inhibits IL-2 synthesis in human T-lymphocytes, an effect that is secondary to a rise in intracellular cyclic AMP concentration [78]. At high concentrations theophylline inhibits proliferation in CD4^+^ and CD8^+ ^cells, an effect that is mediated *via* inhibition of PDE4 [79]. Theophylline also inhibits the chemotactic response of T-lymphocytes, an effect that is also mediated through PDE inhibition [80]. In allergen-induced airway inflammation in guinea pigs, theophylline has a significant inhibitory effect on eosinophil infiltration [81], suggesting that it may inhibit the T-cell-derived cytokines responsible for this eosinophilic response. Theophylline has been reported to decrease circulating concentrations of IL-4 and IL-5 in asthmatic patients [82]. In asthmatic patients low dose theophylline treatment results in an increase in activated circulating CD4^+^ and CD8^+^ T-cells, but a decrease in these cells in the airways, suggesting that it may reduce the trafficking of activated T cells into the airways [83]. This is supported by studies in allergen challenge, where low dose theophylline decreases the number of activated CD4^+ ^and CD8^+^ T-cells in bronchoalveolar lavage fluid after allergen challenge and this is mirrored by an increase in these cells in peripheral blood [84]. These effects are seen even in patients treated with high doses of inhaled corticosteroids, indicating that the molecular effects of theophylline are likely to be different from those of corticosteroids. Theophylline induces apoptosis of T-lymphocytes, thus reducing their survival [32]. This effect may be mediated *via* PDE4 inhibition, so may not be relevant to clinical doses of theophylline. The therapeutic range of theophylline was based on measurement of immediate bronchodilatation in response to the acute administration of theophylline [50]. However, it is possible that the non-bronchodilator effects of theophylline, which may reflect some anti-inflammatory or immunomodulatory effect, may be exerted at lower plasma concentrations and that different molecular mechanisms may be involved [85].

### 4.4. Extrapulmonary Effects

It has been suggested that theophylline may exert its effects in asthma *via* some action outside the airways. It may be relevant that theophylline is ineffective when given by inhalation until therapeutic plasma concentrations are achieved [86]. This may indicate that theophylline has effects on cells other than those in the airway. An effect of theophylline which remains controversial is its action on respiratory muscles. Aminophylline increases diaphragmatic contractility and reverses diaphragm fatigue [87]. This effect has not been observed by all investigators but there are doubts about the relevance of these observations to the clinical benefit provided by theophylline [88]. Whether theophylline has any effects on systemic effects or co-morbidities in COPD patients has not yet been established.

## 5. Pharmacokinetics

There is a close relationship between the acute improvement in airway function and serum theophylline concentration. Below 10 mg/L therapeutic effects (at least in terms of rapid improvement in airway function) are small and above 25 mg/L additional benefits are outweighed by side effects, so that the therapeutic range was usually taken as 10–20 mg/L (55–110 µM) [2]. It is now apparent that non-bronchodilator effects of theophylline may be seen at plasma concentrations of <10 mg/L and that clinical benefit may be derived from these lower concentrations of theophylline. This suggests that it may be necessary to redefine the therapeutic range of theophylline based on anti-asthma effect, rather than the acute bronchodilator response that requires a higher plasma concentration. The dose of theophylline required to give therapeutic concentrations varies among patients, largely because of differences in clearance. Theophylline is rapidly and completely absorbed, but there are large inter-individual variations in clearance, due to differences in hepatic metabolism (Table 2). Theophylline is metabolized in the liver by the cytochrome P450 microsomal enzyme system, and a large number of factors may influence hepatic metabolism. Theophylline is predominantly metabolized by the CYP1A2 enzyme, while at higher plasma concentrations CYP2E1 is also involved (89).

**Table 2:** Factors affecting clearance of theophylline.

*Increased Clearance*	*Decreased Clearance*
Enzyme induction (rifampicin, phenobarbitone, ethanol) Smoking (tobacco, marijuana) High protein, low carbohydrate diet Barbecued meat Childhood	Enzyme inhibition (cimetidine, erythromycin, ciprofloxacin, allopurinol, zileuton) Congestive heart failure Liver disease Pneumonia Viral infection Vaccination (immunization) High carbohydrate diet Old age

### 5.1. Increased Clearance

Increased clearance is seen in children (1–16 years), and in cigarette and marijuana smokers. Concurrent administration of phenytoin and phenobarbitone increases activity of P450, resulting in increased metabolic breakdown, so that higher doses may be required. 

### 5.2. Reduced Clearance

Reduced clearance is found in liver disease, pneumonia and heart failure and doses need to be reduced to half and plasma levels monitored carefully. Decreased clearance is also seen with certain drugs, including erythromycin, certain quinolone antibiotics (ciprofloxacin, but not ofloxacin), allopurinol, cimetidine (but not ranitidine), serotonin uptake inhibitors (fluvoxamine) and the 5-lipoxygenase inhibitor zileuton, which interfere with CYP 1A2 function. Thus, if a patient on maintenance theophylline requires a course of erythromycin, the dose of theophylline should be halved. Viral infections and vaccinations (immunizations) may also reduce clearance, and this may be particularly important in children. Because of these variations in clearance, individualization of theophylline dosage is required and plasma concentrations should be measured 4 h after the last dose with slow-release preparations, when steady state has usually been achieved. There is no significant circadian variation in theophylline metabolism [90].

## 6. Routes of Administration

### 6.1. Intravenous

Intravenous aminophylline has been used for many years in the treatment of acute severe asthma. The recommended dose is now 6 mg/kg given intravenously over 20–30 min, followed by a maintenance dose of 0.5 mg/kg/h. If the patient is already taking theophylline, or there are any factors which decrease clearance, these doses should be halved and the plasma level checked more frequently.

### 6.2. Oral

Plain theophylline tablets or elixir, which are rapidly absorbed, give wide fluctuations in plasma levels and are not recommended. Several effective sustained-release preparations now available are absorbed at a constant rate and provide steady plasma concentrations over a 12–24 h period. Although there are differences between preparations, these are relatively minor and of no clinical significance. Both slow-release aminophylline and theophylline are available and are equally effective (although the ethylene diamine component of aminophylline has very occasionally been implicated in allergic reactions). For continuous treatment, twice daily therapy (approximately 8 mg/kg twice daily) is needed, although some preparations are designed for once daily administration. For nocturnal asthma, a single dose of slow-release theophylline at night may be effective [91,92]. Once optimal doses have been determined plasma concentrations usually remain stable, providing no factors which alter clearance are introduced.

### 6.3. Other Routes

Aminophylline may be given as a suppository, but rectal absorption is unreliable and proctitis may occur, so this route should be avoided. Inhalation of theophylline is irritating and ineffective [86]. Intramuscular injections of theophylline are very painful and should never be given.

## 7. Clinical Use

### 7.1. Acute Exacerbations

Intravenous aminophylline has been used in the management of acute severe asthma for over 50 years, but this use has been questioned in view of the risk of adverse effects compared with nebulized ß_2_-agonists. In patients with acute asthma, intravenous aminophylline is less effective than nebulized ß_2_-agonists [93], and should therefore be reserved for those who fail to respond to ß-agonists. In a meta-analysis of 27 studies which looked at addition of iv aminophylline to nebulized β_2_-agonists, there is no evidence for significant benefit in adults [94] or children [95]. This indicates that aminophylline should not be added routinely to nebulized ß-agonists. Indeed, addition of aminophylline only increases adverse effects. Several deaths have been reported after intravenous aminophylline. In one study of 43 asthma deaths in southern England there was a significantly greater frequency of toxic theophylline concentrations (21%) compared with matched controls (7%) [96]. These concerns have lead to the view that intravenous aminophylline should be reserved for the few patients with acute severe asthma who fail to show a satisfactory response to nebulized β_2_-agonists. When intravenous aminophylline is used it should be given as a slow intravenous infusion with careful monitoring of vital signs and plasma theophylline concentrations should be measured prior to and after infusion. Aminophylline similarly has no place in the routine management of COPD exacerbations [97,98].

### 7.2. Chronic Asthma

In most guidelines for asthma management theophylline is recommended as an additional bronchodilator if asthma remains difficult to control after high doses of inhaled corticosteroids. The introduction of long-acting inhaled β_2_-agonists has further threatened the position of theophylline, since the side effects of these agents may be less frequent that those associated with theophylline and long-acting inhaled β_2_-agonists are more effective controllers than theophylline [99]. Whether theophylline has some additional benefit over its bronchodilator action is now an important consideration. In chronic asthma oral theophylline provides additional control of asthma symptoms even in patients talking regular inhaled steroids [100]. In an uncontrolled study a group of adolescent patients with severe asthma who were controlled with oral and inhaled steroids, nebulized β_2_-agonists, inhaled anticholinergics and sodium cromoglycate, in addition to regular oral theophylline, withdrawal of the oral theophylline resulted in a marked deterioration of asthma control which could not be controlled by further increase in steroids and only responded to reintroduction of theophylline [101]. This suggests that there may be a group of severe asthmatic patients who particularly benefit from theophylline. In a controlled trial of theophylline withdrawal in patients with severe asthma controlled only on high doses of inhaled corticosteroids, there was a significant deterioration in symptoms and lung function when placebo was substituted for the relatively low maintenance dose of theophylline [83]. There is also evidence that addition of theophylline improves asthma control to a greater extent than β_2_-agonists in patients with severe asthma treated with high dose inhaled steroids [102]. This suggests that theophylline may have a useful place in the optimal management of moderate to severe asthma and appears to provide additional control above that provided by high dose inhaled steroids [103,104]. 

Theophylline may be a useful treatment for nocturnal asthma and a single dose of a slow release theophylline preparation given at night may provide effective control of nocturnal asthma symptoms [91,92]. Theophylline has equal efficacy to salmeterol in controlling nocturnal asthma, but the quality of sleep is better with salmeterol compared to theophylline [105]. The mechanism of action of theophylline in nocturnal asthma may involve more than long-lasting bronchodilatation, and could involve inhibition of some components of the inflammatory response, which may increase at night [69]. 

### 7.3. Add-on Therapy

Several studies have demonstrated that adding low dose theophylline to inhaled corticosteroids in patients who are not controlled gives better asthma control than doubling the dose of inhaled corticosteroids. This has been demonstrated in patients with moderate to severe and mild asthma [106,107,108]. Interestingly, there is a greater degree of improvement in forced vital capacity than in FEV_1_, possibly indicating an effect on peripheral airways. Since the improvement in lung function was relatively slow, this suggests that the effect of the added theophylline may be having an anti-inflammatory rather than a bronchodilator effect, particularly as the plasma concentration of theophylline in these studies was < 10 mg/L. These studies suggest that low dose theophylline may be preferable to increasing the dose of inhaled steroids when asthma is not controlled on moderate doses of inhaled steroids; such a therapeutic approach would be much less expensive than adding long-acting inhaled β_2_-agonists. However, theophylline is a less effective option than adding a long-acting inhaled β_2_-agonist [99]. Low dose theophylline is also effective in smoking asthmatics, who have a poor response to inhaled steroids and this may through increasing HDAC2 activity, which is reduced in the airways asthmatic patients who smoke [109].

### 7.4. COPD

Theophylline may also benefit patients with COPD and increases exercise tolerance [110,111]. Theophylline reduces air trapping, suggesting an effect on peripheral airways, and this may explain why some patients with COPD may obtain considerable symptomatic improvement without any increase in spirometric values [112]. The demonstration that low doses of theophylline reduces neutrophils in induced sputum of patients with COPD suggests that theophylline may have some anti-inflammatory effect [71,113]. In COPD macrophages *in vitro* theophylline restores HDAC activity to normal and thus reverses corticosteroid resistance [40]. It also reduces nitrative stress in macrophages from patients with COPD, whereas high doses of an inhaled corticosteroid are without effect [45]. Low dose theophylline increases the recovery from an acute exacerbation of COPD and this is associated with reduced inflammation and increased HDAC activity [114]. This suggests that corticosteroids may be useful in reversing corticosteroid resistance in patients with COPD, but long-term clinical trials are now needed to confirm this [115,116].

### 7.5. Interaction with ß_2_-Agonists

If theophylline exerts its effects by PDE inhibition, a synergistic interaction with ß-agonists would be expected. Many studies have investigated this possibility, but while there is good evidence that theophylline and ß-agonists have additive effects, true synergy is not seen. β_2_-Agonists may cause relaxation of airway smooth muscle *via* several mechanisms. Classically, they increase intracellular cyclic AMP concentrations, which were believed to be an essential event in the relaxation response. However, β_2_-agonists cause bronchodilatation, at least in part, by opening maxi-K channels in airway smooth muscle cells [48]. Maxi-K channels are opened by low concentrations of ß_2_-agonists which are likely to be therapeutically relevant. β_2_-Receptors may be coupled directly to maxi-K channels *via* the α-subunit of G_s_ and therefore may induce relaxation without any increase in cyclic AMP, thus accounting for a lack of synergy [117]. 

Repeated administration of β_2_-agonists may result in tolerance; however, this may be explained by down-regulation of β_2_-receptors, an additional mechanism may involve up-regulation of PDE enzymes (especially PDE4D) which then break down cyclic AMP more readily [118]. Theophylline may therefore theoretically prevent the development of tolerance. However, in a clinical study theophylline failed to prevent the development of tolerance to the bronchoprotective effect of salmeterol in asthmatic patients [119].

## 8. Side Effects

The main limitation to the use of theophylline in conventional doses has been the high frequency of adverse effects [120]. Unwanted effects of theophylline are usually related to plasma concentration and tend to occur when plasma levels exceed 20 mg/L; however, some patients develop side-effects even at low plasma concentrations. To some extent side effects may be reduced by gradually increasing the dose until therapeutic concentrations are achieved.

The commonest side effects are headache, nausea and vomiting, abdominal discomfort and restlessness. There may also be increased acid secretion, gastroesophageal reflux and diuresis. At high concentrations convulsions and cardiac arrhythmias may occur and, as stated previously, there is concern that intravenous aminophylline administered in the emergency room may be a contributory factor to the deaths of some patients with severe asthma [96].

Some of the side-effects of theophylline (central stimulation, gastric secretion, diuresis and arrhythmias) may be due to adenosine receptor antagonism (A_1A _receptors) and these may therefore be avoided by PDE inhibitors or by doxifylline when it is available, as discussed above. The commonest side effects of theophylline are nausea and headaches, which may be due to inhibition of certain PDEs (e.g., PDE4 in the vomiting center) and cardiac arrhythmias due to inhibition of PDE3 [121].

## 9. Future Developments

Although theophylline has recently been used much less in developed countries, there are reasons for thinking that it may come back in fashion for the treatment of severe asthma, smoking asthma and COPD, with the recognition that it may have an anti-inflammatory and immunomodulatory effect when given in low doses (plasma concentration 5–10 mg/L) [3]. At these low doses the drug is easier to use, side effects are uncommon and the problems of drug interaction are less of a problem, thus making the clinical use of theophylline less complicated. Theophylline appears to have an effect that is different from those of corticosteroids and it may therefore be a useful drug to combine with low dose inhaled steroids. The molecular mechanism of anti-inflammatory effects of theophylline is now becoming clearer and it seems likely that there is a synergistic interaction with the anti-inflammatory mechanism of corticosteroids through restoration of HDAC activity. This interaction may underlie the beneficial effects of theophylline when added to inhaled corticosteroids. This may be particularly appropriate in patients with more severe asthma in whom corticosteroids are less effective as there may be a reduction in HDAC activity in these patients [37] as well as in smoking asthmatics patients [109] and patients with COPD [114]. As slow-release theophylline preparations are cheaper than long-acting inhaled β_2_-agonists and leukotriene modifiers, this may justify the choice of low dose theophylline as the add-on therapy for asthma control. In addition, compliance with oral therapy is likely to be greater than with inhaled therapies [122]. This suggests that low dose theophylline may find an important place in the management in patients of patients with severe asthma and all patients with COPD. 
